# Social vulnerability and the risk of respiratory virus infection in households: a case-ascertained study

**DOI:** 10.1186/s12879-025-12310-6

**Published:** 2025-12-13

**Authors:** Sara H. Goodman, Alexandra Mellis, Clea C. Sarnquist, Carlos G. Grijalva, H. Keipp Talbot, Melissa S. Stockwell, Son H. McLaren, Ellen Sano, Suchitra Rao, Edwin Asturias, Huong Q. Nguyen, Edward A. Belongia, Katherine D. Ellingson, Karen Lutrick, Natalie M. Bowman, Jessica T. Lin, Melissa A. Rolfes, Jessica E. Biddle, Sarah E. Smith-Jeffcoat, Yvonne Maldonado

**Affiliations:** 1 Department of Pediatrics, Stanford University School of Medicine, Stanford, CA USA; 2 Centers for Disease Control and Prevention, Atlanta, GA USA; 3 Vanderbilt University Medical Center, Nashville, TN USA; 4 Departments of Pediatrics and Population and Family Health, Columbia University Irving Medical Center, New York-Presbyterian Hospital, New York, NY USA; 5 Department of Emergency Medicine, Columbia University Irving Medical Center, New York-Presbyterian Hospital, New York, NY USA; 6 University of Colorado School of Medicine, Aurora, CO USA; 7 Marshfield Clinic Research Institute, Marshfield, WI USA; 8 University of Arizona Mel and Enid Zuckerman College of Public Health, Tucson, AZ USA; 9 University of North Carolina at Chapel Hill, Chapel Hill, NC USA

**Keywords:** SARS CoV-2, Influenza, Epidemiology, Public Health, Household infection, Case-ascertained study, Social vulnerability index, Social epidemiology

## Abstract

**Objectives:**

This study examines whether the Social Vulnerability Index (SVI), a location-based composite measure of social vulnerability, is associated with the risk of SARS-CoV-2 or influenza infection within households.

**Study design:**

Prospective cohort case-ascertained household transmission study.

**Methods:**

We analyzed data from a case-ascertained household transmission study conducted across multiple U.S. sites (September 2021–May 2023). Household contacts of index cases with confirmed infections self-collected nasal swabs daily for ten days, tested via RT-PCR for SARS-CoV-2 or influenza. Age, sex, and vaccine receipt were self-reported, with vaccination verified. Household addresses were geocoded to 2020 census tracts and linked to the national SVI percentile. Using modified Poisson regression models with generalized estimating equations, we assessed associations between census tract-level SVI and infection risk, adjusting for age, sex, vaccine receipt, and clustering by census tract. Participants included household contacts of index cases with SARS-CoV-2 (793 households, 1,408 participants) or influenza (273 households, 512 participants).

**Results:**

We found that higher overall SVI was associated with increased SARS-CoV-2 infection risk (adjusted Incidence risk ratio [aIRR] 1.24; 95% CI: 1.00, 1.52). Specifically, the socioeconomic SVI domain was linked to higher infection risk (aIRR = **1.24**; 95% CI: **1.02**,** 1.51**). Other SVI domains were not statistically significant. For influenza, SVI was overall associated with greater infection, but confidence intervals crossed the null (aIRR = 1.45; 95% CI: 0.88, 2.39).

**Conclusions:**

Household contacts of SARS-CoV-2 index cases in high-SVI areas faced an increased risk of infection. No significant association was found for influenza, likely due to the small sample size. Increased access to SARS-CoV-2 testing, treatment, and preventive measures (e.g., masking, handwashing, isolation) may be especially important in high-SVI areas.

**Supplementary Information:**

The online version contains supplementary material available at 10.1186/s12879-025-12310-6.

## Introduction

Socioeconomic status, demographic, and geographic factors can facilitate the spread of respiratory viruses, including SARS-CoV-2 and influenza [1, 2]. Understanding the role of these social factors in respiratory virus transmission informs public health strategies to control the spread of respiratory infections.

Social vulnerability involves multiple risk factors potentially associated with infection risk, such as socioeconomic status, household characteristics, race/ethnicity, household type/crowding, and transportation. Many studies have consistently illustrated an association between social vulnerability and adverse health outcomes, such as respiratory virus infection [2–8]. 

A substantial proportion of SARS-CoV-2 and influenza transmission is thought to occur within households [9]. Yet, to date, no studies have examined the influence of social vulnerability at the census tract level and the risk of SARS-CoV-2 or influenza transmission within the household [1, 2, 7, 8, 10–13]. To fill these knowledge gaps, we examined if a higher Social Vulnerability Index (SVI) percentile, and therefore increased census-tract level social vulnerability, was associated with an increased risk of SARS-CoV-2 or influenza infection among household contacts of an index case.

## Methods

This analysis used data from the Respiratory Virus Transmission Study (RVTN) project, a CDC-sponsored prospective case-ascertained household transmission study conducted across seven sites in the United States between September 2021 and May 2023 [14]. The SARS-CoV-2 variants that were circulating this time were primarily Delta, Omicron, and XBB [15, 16]. 

### Study population

We defined a household contact as an individual who resided in the same household as the index case. Those who lived in a congregated setting such as a nursing home or dormitory were ineligible [14, 17]. Eligibility criteria for participation in this study included that participants were household contacts of an index case who tested positive for SARS-CoV-2 or influenza within seven days of symptom onset or five days after presentation at the clinic, and that at least two-thirds of household members agreed to enroll in the study (supplemental Figs. 1 and 2)and [14, 17].

## Data collection

Participants collected nasal swabs daily for ten days and were tested for SARS-CoV-2 or influenza via reverse transcription-polymerase chain reaction (RT-PCR) [14]. Demographic and household characteristics were self-reported at the time of enrollment. Vaccine receipt was self-reported and verified by reviewing medical records, vaccination registries, vaccination cards, and records from pharmacies and other non-traditional sources as appropriate [14]. 

**Fig. 1:**
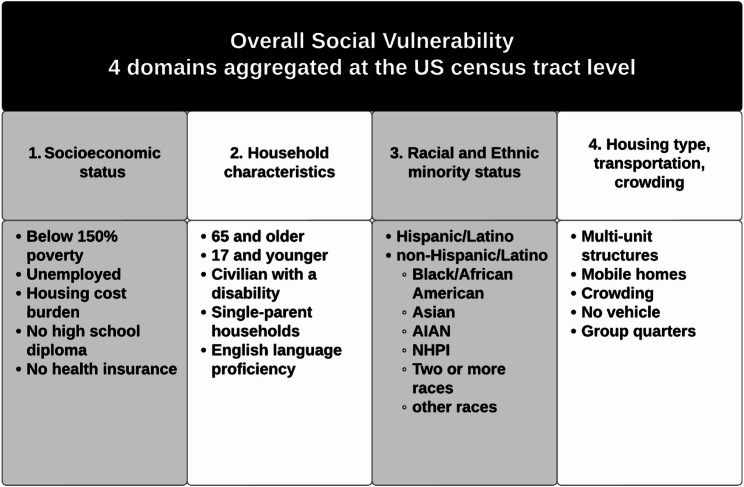
2020 Social vulnerability index domains and domain components^1^ CDC/ATSDR Social Vulnerability Index (SVI). Published November 16, 2022. Accessed May 12, 2023. https://www.atsdr.cdc.gov/place-health/php/svi/index.html

The Social Vulnerability Index (SVI) is a location-based estimate of vulnerability to natural or human-made disasters developed by the Centers for Disease Control and Prevention (CDC) and the Agency for Toxic Substances and Disease Registry (ATSDR) [1, 2, 4, 7, 8, 10–13]. The SVI uses responses from 16 questions on the American Community Survey (ACS) to provide overall and domain-based estimates of social vulnerability relative to other locations [18]. The four SVI domains include (a) socioeconomic status, (b) household characteristics, (c) racial and ethnic minority status, and (d) housing type, transportation, and crowding (Fig. 1) [4]. A higher SVI percentile indicates a higher level of vulnerability [3]. SVI is calculated for each U.S. census tract. Census tracts are subdivisions of counties, smaller than ZIP codes, with populations ranging from 1,000 to 8,000 residents, averaging 4,000 people, and remain stable across decennial censuses [19]. 

To understand the effects of the 2020 SVI on SARS-CoV-2 and influenza household transmission, we geocoded and mapped households to 619 2020 United States decennial census tracts and their corresponding SVI percentile using the United States Census Bureau geocoder [19]. Enrolled geocoded households were spatially joined to SVI at the census tract level with the national SVI percentile among (a) the SVI index percentile overall and (b) the four domains discussed above [4]. The national SVI percentile represents census tracts by percentile ranking relative to the rest of the United States on a scale from 0 to 1, with one being the most vulnerable [20]. 

### Data analysis

As this analysis aims to understand the risk of household infection, only household contacts were included. Participants provided at least three days of follow-up with complete symptom diaries and valid tests. Household contacts were excluded if they were missing vaccination information for SARS-CoV-2 (among SARS-CoV-2 household contacts) or influenza (among influenza household contacts), if there was co-detection of SARS-CoV-2 and influenza in the index case, or if the household could not be geocoded to a 2020 census tract.

Our primary outcome of interest was infection among household contacts, as verified by a positive RT-PCR test for SARS-CoV-2 or influenza 10 days after study enrollment, with each virus analyzed separately [14]. Our exposure of interest was the SVI. We analyzed SVI as its overall percentile and by each of the four SVI domain percentiles. We analyzed these percentiles as continuous variables with values between 0 and 1, including all participants and their SVI values when calculating crude and adjusted incidence risk ratios [21]. Additional participant characteristics of interest were sex (male, female, unknown), enrollment site, age in years as a continuous variable, and vaccine receipt for SARS-CoV-2 and influenza. We defined individuals as SARS-CoV-2 vaccinated if they had received two or more doses of a COVID-19 vaccine 14 days or more before the earliest illness onset in the household and unvaccinated if they received less than two doses. We defined influenza vaccination as participant receipt of at least one dose of the current season’s influenza vaccine (2021-22 for participants enrolled before September 1 of 2022, and 2022-23 for participants enrolled thereafter), received 14 days or more before the earliest illness onset in the household. Participants were followed up from symptom onset through the earliest instance of PCR result, withdrawal date, end of follow up, or last daily symptom diary [17, 22]. 

We calculated descriptive statistics, including frequencies of categorical variables and measures of central tendency, and median and interquartile range for continuous variables. We performed crude, bivariable regressions, clustering by household census tract and robust standard errors to understand if the SVI percentile was associated with the risk of infection in five models (overall SVI and each domain) that used generalized estimating equations with a modified Poisson regression model (primary results). We repeated these analyses with both national and state-level SVI percentiles to understand whether the effect of SVI on infection risks differed when considering local relative vulnerability rather than broader, national-level vulnerability. To understand whether the effect of SVI on infection risks was changed by inclusion of individual-level characteristics of age, sex, site, or race-ethnicity, we repeated adjusted regressions accounting for these terms. For each virus separately, we also explored vaccination as a mediator of the relationship between social vulnerability and infection risks. All analyses were performed using R Statistical Software (v4.2.2; R Core Team 2023) using the geepack and GEEMediate R packages [23–26]. 

## Ethical considerations

This study was reviewed and approved by the Vanderbilt University Medical Center’s IRB (See 45 C.F.R. part 46.114; 21 C.F.R. part 56.114).

## Results

### Demographic characteristics of SARS-CoV-2 household contacts

Among 1408 household contacts from 793 SARS-CoV-2-affected households, 66.3% (*n* = 934) tested positive for SARS-CoV-2 (Table 1). The median household size was four household members (Interquartile range (IQR) = 3–4). Nearly a third of household contacts were under 18 years old (30%; *n* = 422), and 47% were between 18 and 49 years old (*n* = 664). Most participants were female (53.6%; *n* = 755), 55.6% were white, non-Hispanic (*n* = 769),). Two-thirds (66.9%) of household contacts (*n* = 942) lived in single-family residences. Over three-fourths of household contacts (76.6%; *n* = 1078) were vaccinated against SARS-CoV-2, leaving only 23.4% of unvaccinated household contacts. The median overall SVI percentile was 0.40 (IQR: 0.15–0.80) among SARS-CoV-2 household contacts (Table 2).

**Table 1:** Characteristics of household contacts exposed to SARS-CoV-2 or influenza enrolled in a case ascertained household transmission study, united States, September 2021-May 2023

Characteristic	SARS-CoV-2 Household Contacts, *N* = 1,408	Influenza Household Contacts, *N* = 512
	n (%)	n (%)
Test result^1^		
Negative	474 (33.7%)	235 (45.9%)
Positive	934 (66.3%)	277 (54.1%)
Household size		
mean	2.48	4.32
median (IQR)	3 (3–4)	4 (3–5)
Age group, years		
0–4	106 (7.5%)	37 (7.2%)
5–11	157 (11.2%)	89 (17.4%)
12–17	159 (11.3%)	64 (12.5%)
18–49	664 (47.2%)	262 (51.2%)
50–64	213 (15.1%)	41 (8.0%)
65+	109 (7.7%)	19 (3.7%)
Enrollment site		
Arizona (AZ)	151 (10.7%)	5 (1.0%)
Colorado (CO)	80 (5.7%)	3 (0.6%)
Columbia (NY)	292 (20.7%)	279 (54.5%)
Marshfield (WI)	135 (9.6%)	6 (1.2%)
Nashville (TN)	515 (36.6%)	180 (35.2%)
North Carolina (NC)	105 (7.5%)	28 (5.5%)
Stanford (CA)	130 (9.2%)	11 (2.2%)
Sex		
Female	755 (53.6%)	315 (61.5%)
Male	647 (45.9%)	197 (38.5%)
unknown	6 (0.4%)	0 (0%)
Race/ethnicity		
White, non-Hispanic	769 (55.6%)	137 (26.8%)
Hispanic/Latino	407 (28.9%)	278 (54.5%)
Black, non-Hispanic	88 (6.3%)	60 (11.8%)
Asian, non-Hispanic	55 (3.9%)	7 (1.4%)
Multiple races, non-Hispanic	43 (3.1%)	6 (1.2%)
Unknown/Refused	18 (1.3%)	19 (3.7%)
Native Hawaiian/Other Pacific Islander, non-Hispanic	4 (0.3%)	1 (0.2%)
Missing	24 (1.7%)	2 (0.4%)
American Indian/Alaska Native, non-Hispanic	0 (0%)	2 (0.4%)
Vaccine receipt^2,3^		
Unvaccinated	330 (23.4%)	307 (60.0%)
Vaccinated	1078 (76.6%)	205 (40.0%)
Education		
Never attended school or only attended kindergarten	132 (9.4%)	44 (8.6%)
Less than a high school education	334 (23.7%)	184 (35.9%)
High school graduate	170 (12.1%)	72 (14.1%)
Some college	221 (15.7%)	62 (12.1%)
College graduate	507 (36.0%)	123 (24.0%)
Don’t know, don’t remember/prefer not to answer/unknown	44 (3.1%)	25 (4.9%)
Household type		
Apartment/Condo	410 (29.1%)	301 (58.8%)
Duplex/townhome	56 (4.0%)	7 (1.4%)
Single-family home	942 (66.9%)	204 (39.8%)

^1^ Household contacts were tested for the pathogen to which they were known to be exposed i.e. household contacts of an index case who tested positive for SARS-CoV-2 were tested for SARS-CoV-2 and household contacts of an index case who tested positive for influenza were tested for influenza over the course of 10 days

^2^ SARS-CoV-2 vaccine receipt defined as unvaccinated if received 1 or fewer COVID-19 vaccine doses and vaccinated if received 2 or more COVID-19 vaccine doses. Influenza vaccine receipt defined as unvaccinated if did not receive annual influenza vaccine and vaccinated if participant did receive the seasonal influenza vaccine

^3^ Participants that were < 6 months of age were not eligible for COVID-19 and influenza vaccines

**Table 2:** Overall and domain-based SVI percentiles at the census tract level among household contacts exposed to SARS-CoV-2 or influenza by vaccine receipt^1,2^, united States, September 2021-May 2023

	SARS-CoV-2 household contact vaccine receipt			Influenza household contact vaccine receipt		
	Unvaccinated (*N* = 330)	Vaccinated (*N* = 1078)	Overall (*N* = 1408)	Unvaccinated (*N* = 307)	Vaccinated (*N* = 205)	Overall (*N* = 512)
Overall SVI Median (IQR)	0.57 (0.27–0.95)	0.36(0.13–0.72)	0.40 (0.15–0.8)	0.84 (0.29–0.98)	0.79 (0.18–0.97)	0.83 (0.26–0.98)
Socioeconomic status domain Median (IQR)	0.60 (0.24–0.9)	0.31 (0.12–0.69)	0.34 (0.14–0.77)	0.82 (0.46–0.93)	0.73 (0.19–0.92)	0.80 (0.27–0.93)
Household characteristics domain Median (IQR)	0.57 (0.28–0.87)	0.40 (0.17–0.68)	0.42 (0.18–0.74)	0.64 (0.3–0.91)	0.58 (0.26–0.84)	0.62 (0.28–0.90)
Racial/ethnic minorities domain Median (IQR)	0.56 (0.29–0.92)	0.48 (0.28–0.75)	0.5 (0.28–0.79)	0.86 (0.48–0.95)	0.77 (0.33–0.94)	0.84 (0.36–0.95)
Household type and transportation domain Median (IQR)	0.68 (0.36–0.94)	0.5 (0.17–0.78)	0.56 (0.19–0.83)	0.87 (0.45–0.95)	0.75 (0.18–0.95)	0.85 (0.32–0.95)

^1^ SARS-CoV-2 vaccine receipt defined as unvaccinated if received 1 or fewer COVID-19 vaccine doses and vaccinated if received 2 or more COVID-19 vaccine doses. Influenza vaccine receipt defined as unvaccinated if did not receive annual influenza vaccine and vaccinated if participant did receive the seasonal influenza vaccine

^2^ Participants that were < 6 months of age were not eligible for COVID-19 and influenza vaccines

### Demographic characteristics of influenza household contacts

Among 512 household contacts from 273 influenza-affected households, 54.1% (*n* = 277) tested positive for influenza (Table 1). Individuals in our influenza cohort identified as predominantly Hispanic/Latino (*n* = 278 (54.5%)). The median household size was four household members (IQR = 3–5). Approximately 37% of household contacts were under age 18 (*n* = 190). Almost 59% of household contacts (*n* = 301) lived in apartments or condominiums, while 39.8% lived in single-family homes. 40% of household contacts (*n* = 205) were vaccinated against influenza. The median overall SVI percentile was 0.83 (IQR: 0.26–0.98) among influenza household contacts (Table 2).

## SVI and vaccine receipt^1^

Among SARS-CoV-2 household contacts, those who were vaccinated against SARS-CoV-2 had lower median overall SVI (0.36; IQR: 0.13–0.72) compared to unvaccinated household contacts (0.57; IQR: 0.27–0.95). This trend was similar across all four SVI domains. Similarly, among influenza household contacts, those who were vaccinated against influenza had lower median overall SVI (0.79; IQR: 0.18–0.97) compared to unvaccinated participants (0.84; IQR: 0.29–0.98; Table 2, supplemental Figs. 3–4).

### SVI and risk of SARS-CoV-2 infection

The risk of SARS-CoV-2 infection was higher among household contacts with a higher SVI (crude incidence risk ratio [cIRR]: 1.20; 95% CI: 1.04–1.38; Table 3), consistent with a 20% increased risk of infection among household contacts with an overall SVI percentile of 1 (most vulnerable) compared to those with an SVI percentile of 0 (least vulnerable). Similarly, the risk of SARS-CoV-2 infection was higher among household contacts with a higher socioeconomic domain SVI percentile, consistent with a 24% increased risk of infection (cIRR = 1.24; 95% CI: 1.08–1.43) and household contacts with a higher racial/ethnic minority domain SVI percentile, consistent with a 25% increased risk of infection (cIRR = 1.25; 95% CI: 1.05–1.48). The household characteristics domain and the household type/transportation/crowding domain SVI percentiles were not statistically associated with an increased risk of infection. After adjusting for vaccine status as well as age sex, and study site, the adjusted incidence risk ratios (aIRRs) of SARS-CoV-2 infection (Table 3) remained significant for SVI overall (aIRR = 1.24; 95% CI: 1.00-1.52), and socioeconomic status (aIRR = 1.24; 95% CI: 1.02–1.51) domains, however these estimates pushed closer to null values when adjusted for vaccination status. The point estimate for the relationship between the racial/ethnic minority domain shifted toward higher risk, but the confidence intervals widened and crossed the null (aIRR = 1.34, 95% CI: 0.98–1.82). As seen in the crude results, we did not see significant IRRs for the household characteristic percentile. We found no significant mediation effect of SARS-CoV-2 vaccination on the relationship between SVI and SARS-CoV-2 infection (supplemental Table 3).

**Table 3:** Crude (cIRR) and adjusted incidence risk ratios (aIRRs)^1^ of SARS-CoV-2 or influenza among exposed household contacts by social vulnerability index overall and by domain percentiles, cluster adjusted within census tract and in the adjusted rates by vaccine status^2,3^ in a case ascertained household transmission study, united States, September 2021-May 2023

	SARS-CoV-2 Households 793 households, 1408 participants						Influenza Households 273 households, 512 participants					
**Characteristic**	**Crude IRR** ^*1*^	**95% CI** ^*1*^		**Adjusted IRR** ^*1*^	**95% CI** ^*1*^		**Crude IRR** ^*1*^	**95% CI** ^*1*^		**Adjusted IRR** ^*1*^	**95% CI** ^*1*^	
Overall SVI percentile	**1.20**	**1.04**,** 1.38**		**1.24**	**1.00**,** 1.52**		1.18	0.89, 1.57		1.45	0.88, 2.39	
Socioeconomic domain SVI percentile	**1.24**	**1.08**,** 1.43**		**1.24**	**1.02**,** 1.51**		1.15	0.85, 1.55		1.26	0.74, 2.12	
Household Characteristics domain SVI percentile	1.11	0.95, 1.30		1.08	0.88, 1.33		1.32	0.96, 1.83		1.52	0.98, 2.35	
Racial/ethnic minorities domain SVI percentile	**1.25**	**1.05**,** 1.48**		1.34	0.98, 1.82		1.26	0.88, 1.79		1.90	0.95, 3.80	
Household type, transportation, and crowding domain SVI percentile	1.12	0.97, 1.30		1.11	0.92, 1.35		1.15	0.85, 1.56		1.31	0.81, 2.12	

^*1*^IRR = Incidence risk ratio, CI = Confidence Interval

^1^ Adjusted models included vaccine receipt, and clustering by census tract

^2^ SARS-CoV-2 vaccine receipt defined as unvaccinated if received 1 or fewer COVID-19 vaccine doses and vaccinated if received 2 or more COVID-19 vaccine doses. Influenza vaccine receipt defined as unvaccinated if did not receive annual influenza vaccine and vaccinated if participant did receive the seasonal influenza vaccine

^3^ Participants that were < 6 months of age were not eligible for COVID-19 and influenza vaccines

### SVI and risk of influenza infection

There was not a statistically significant association between household infections of influenza household contacts with an overall SVI percentile of 1 (highest SVI percentile) compared to those with an SVI percentile of 0 (lowest SVI percentile) (cIRR: 1.18; 95% CI: 0.89–1.57; Table 3). After adjusting for vaccination, age, sex, and study site, we did not see a statistically significant association between SVI and household infections (aIRR 1.45; 95% CI: 0.88–2.39; Table 3). Similar to the SARS-CoV-2 households, we found no significant mediation effect of vaccine receipt on the relationship between SVI and influenza infection risk (supplemental Table 4).

## Discussion

To our knowledge, this is the first case-ascertained study to examine SVI and its association with both influenza and SARS-CoV-2 risk of household infection at the census tract level. After adjusting for age, sex, study site, COVID-19 vaccine receipt, and clustering by census tract, we saw the persistent effects of social vulnerability on SARS-CoV-2 infection among household contacts, mainly reflected in the SVI overall percentile, the socioeconomic domain percentile, and the racial/ethnic minority domain percentile. This is consistent with the literature, where higher SVI was associated with increased rates of SARS-CoV-2 infection in households [1]. 

Several studies examined the association between SVI and influenza infection. One study looked at SVI and its association with the proportion of Medicare recipients with influenza vaccination [10]. Using 2018 SVI data, investigators found a 10% increase in SVI percentile, which was inversely associated with a 0.87 unit decrease in the proportion of vaccinated individuals in the county [10]. This meant that people living in counties with a higher SVI percentile (more vulnerable) were less likely to be vaccinated. Another study examined influenza hospitalization rates by race/ethnicity using the Influenza Hospitalization Surveillance Network (FluSurv-NET) data merged with 2020 SVI data [2]. After adjusting for SVI among hospitalized influenza patients, researchers found that people who identified as African American had higher hospitalization rates (1.6–1.8 times higher) when compared to influenza hospitalizations among people who identified as white [2]. This study showed the lingering effects of race/ethnicity on influenza hospitalizations after adjusting for social vulnerability, implying that SVI does not explain these differences alone.

Our results reinforce the influence of social factors on public health [10], on SARS-CoV-2 infection, specifically, and bolster the importance of understanding race/ethnicity and socioeconomic status as risk factors for infectious respiratory viruses [3]. For example, studies at the beginning of the COVID-19 pandemic (which did not examine SVI) found that individuals who identified as African American and individuals who identified as Hispanic/Latino were disproportionately affected by SARS-CoV-2 [27], likely because they were more likely to be essential workers (in health care or the service industry,) increasing their virus exposure [28]. Once the virus was introduced in the household, studies suggest individuals with lower socioeconomic status or living in a neighborhood made up of individuals identifying as predominantly minority races/ethnicities may have reduced access to health-related resources [3, 29]. Examples include inadequate air filtration/ventilation, insufficient cleaning supplies, lower health literacy/health education, and the inability to isolate infected family members due to crowded living conditions [30]. 

In our study population, we saw lower SVI percentiles by both influenza and COVID-19 vaccination receipt; vaccinated individuals lived in lower SVI percentile areas overall compared to unvaccinated individuals. Vaccination did not erase the effect of SVI on household infection overall. Other interventions for these populations may include increasing access to healthcare with neighborhood-based programs and reducing patient care costs. A systematic review examined SVI and SARS-CoV-2 transmission and advocated that social vulnerability and health equity be included in emergency planning for future pandemics, ensuring accessible interventions for all residents [31]. Equitable interventions may include promoting low-cost, easily accessible nonpharmaceutical interventions such as masking, isolation/separation of ill from healthy people, education on hand hygiene, and minimizing virus spread from coughing and sneezing.

Other studies have found that increased social vulnerability, especially in areas with low socioeconomic status and higher percentages of racial/ethnic minorities, was associated with higher rates of influenza transmission [32–35]. Furthermore, additional studies are needed to examine vaccine uptake among individuals in areas with lower socioeconomic status to support evidence-informed interventions to improve influenza vaccine coverage [7, 10]. 

### Limitations

There are several limitations of this study worth noting. First, the SVI variables are aggregated at the census tract level and may not reflect the actual social vulnerability of individual participants’ households. We are unable to distinguish whether census-tract level vulnerability or individual vulnerability (access to care, distance to care) which could make our participants more or less likely to have household infection. We used the SVI domain percentile at the national level to enable comparability between sites; however, we obtained similar IRR point estimates when using state-level SVI (supplementary figure 5).We also only assessed the effect of SVI as a continuous variable in our sensitivity analysis; we did not find statistical significance using the log or squared form of SVI percentile.

Second, since this was a case-ascertained study, it could be subject to selection bias as participants were not randomly selected, and the index patients were included because they sought care. Also, our results may not be externally valid and may not generalize to other geographical settings that differ in size or population composition from the enrolled study sites. Third, although our influenza household results were not statistically significant, this could be due to the atypical influenza 2021–2022 season with lower influenza cases overall due to COVID-19 pandemic prevention measures such as isolation and masking [36]. The difference in sample sizes of the SARS-CoV-2 cohort (*n* = 1408) and influenza cohort (*n* = 512) may have limited our ability to detect statistically significant differences in the risk of infection in that cohort. This analysis does not rule out potential influence on household influenza transmission. We performed analyses by individual factors (supplemental Tables 1 and 2, supplemental figure 5) for SARS-CoV-2 and influenza; however, including individual factors may have over-adjusted the model because the SVI percentile includes race/ethnicity and age. Finally, we are unable to account for the impact of viral evolution and different prevention measures over the course of the study, as this is beyond the scope of our data.

### Strengths

Our study analyzes SVI at the census tract level. Past studies examined SVI at the county level to assess SARS-CoV-2 public health interventions and county-level vaccine coverage [7, 12, 13]. Researchers found that counties with lower SVI percentiles had greater COVID-19 vaccination coverage than counties with higher SVI percentiles [13]. Dasgupta et al. [1]. examined the association between county-level SVI and SARS-CoV-2 case reporting, finding higher disease reporting rates in counties with higher SVI percentiles [1]. These studies can aid resource allocation at the county level to decrease respiratory virus transmission and adverse health outcomes. However, they lack the granularity of smaller geographic areas such as census tracts [8]. 

### Public health implications

SVI is a versatile index that allows for nationwide and statewide comparisons. This study and prior research show that higher SVI is associated with a higher household infection risk of SARS-CoV-2. Since SVI information is publicly available, it can be used to respond to seasonal and unexpected outbreaks of infectious respiratory viruses. Although we did not see statistically significant results between SVI and influenza, we found similar point estimates in a smaller cohort. Since the prevention methods for both respiratory viruses are similar, these findings could impact household transmission. Additional research is needed with a larger cohort size to test this hypothesis. Households in higher social vulnerability areas are at increased risk of SARS-CoV-2 infection after the pathogen has been introduced into the household. Given that this is a household-level study, quarantining household sick family members is not possible; therefore, we cannot evaluate a strict quarantine policy in this study [37]. In many cases, complete isolation of an infected person in crowded living spaces is impossible. Therefore, increasing access to SARS-CoV-2 testing and treatment to those who are most socially vulnerable and the addition of primary prevention measures (masking, hand hygiene, and covering coughs and sneezes) are key.

## Supplementary Information

Below is the link to the electronic supplementary material.

Supplementary Material 1

## Data Availability

The data supporting this study’s findings are available from the Centers for Disease Control and Prevention, but restrictions apply to the availability of these data. These data were granted permission for use in the current study and so are not publicly available.

## Footnotes

1 infants aged < 6 months are not eligible for COVID-19 or influenza vaccine, however infants that participated in the study were indeed included in this analysis

## Abbreviations

aIRR: Adjusted incidence risk ratio
ASTDR: Agency for Toxic Substances and Disease Registry
CDC: United States Centers for Disease Control and Prevention
CI: Confidence Interval
cIRR: Crude incidence risk ratio
IRB: Institutional Review Board
SARS: CoV–2 = severe acute respiratory syndrome coronavirus 2
SVI: Social Vulnerability Index
