# Supplementary material for: Social vulnerability and the risk of respiratory virus infection in households: a case-ascertained study
Source: BMC Infect Dis. 2025 Dec 13;26:92. doi: 10.1186/s12879-025-12310-6 (PMC12822291; doi:10.1186/s12879-025-12310-6)
Supplement: Supplementary file 1 — Supplementary Material 1 [file 12879_2025_12310_MOESM1_ESM.docx]

Social vulnerability and the risk of respiratory virus infection in households: a case-ascertained study: Supplemental tables and figures

**Supplement**

Supplemental Table 1: Adjusted incidence risk ratios (aIRRs)^1^ of SARS-CoV-2 among exposed household contacts by National Social Vulnerability Index overall and by domain percentiles, adjusted for age, study site, and race/ethnicity, cluster adjusted within census tract in a case ascertained household transmission study, United States, September 2021-May 2023^1^

|  | **Overall** | | **Theme 1: Socioeconomic Status** | | **Theme 2: Household Characteristics** | | **Theme 3: Racial/ethnic minorities** | | **Theme 4: Household type-transportation-crowding** | |
| --- | --- | --- | --- | --- | --- | --- | --- | --- | --- | --- |
| **Characteristic** | **IRR** | **95% CI** | **IRR** | **95% CI** | **IRR** | **95% CI** | **IRR** | **95% CI** | **IRR** | **95% CI** |
| SVI | 1.24 | 1.00, 1.52 | 1.24 | 1.02, 1.51 | 1.08 | 0.88, 1.33 | 1.34 | 0.98, 1.82 | 1.11 | 0.92, 1.35 |
| Age | 1.00 | 1.00, 1.00 | 1.00 | 1.00, 1.00 | 1.00 | 1.00, 1.00 | 1.00 | 1.00, 1.00 | 1.00 | 1.00, 1.00 |
| Sex |  |  |  |  |  |  |  |  |  |  |
| Female | — | — | — | — | — | — | — | — | — | — |
| Male | 0.96 | 0.89, 1.03 | 0.96 | 0.89, 1.03 | 0.95 | 0.89, 1.03 | 0.95 | 0.89, 1.03 | 0.96 | 0.89, 1.03 |
| Unknown | 1.14 | 0.74, 1.75 | 1.14 | 0.75, 1.73 | 1.13 | 0.74, 1.73 | 1.13 | 0.74, 1.73 | 1.14 | 0.74, 1.76 |
| missing | 2.13 | 1.45, 3.12 | 2.12 | 1.45, 3.11 | 2.11 | 1.43, 3.12 | 2.10 | 1.43, 3.10 | 2.08 | 1.41, 3.05 |
| Enrollment site |  |  |  |  |  |  |  |  |  |  |
| Arizona (AZ) | — | — | — | — | — | — | — | — | — | — |
| Colorado (CO) | 1.27 | 0.97, 1.67 | 1.25 | 0.95, 1.63 | 1.24 | 0.94, 1.64 | 1.24 | 0.94, 1.62 | 1.26 | 0.95, 1.65 |
| Columbia (NY) | 1.14 | 0.90, 1.43 | 1.14 | 0.90, 1.43 | 1.19 | 0.94, 1.50 | 1.11 | 0.88, 1.41 | 1.16 | 0.92, 1.48 |
| Marshfield (WI) | 1.09 | 0.82, 1.44 | 1.09 | 0.82, 1.44 | 1.08 | 0.81, 1.43 | 1.18 | 0.87, 1.61 | 1.05 | 0.80, 1.39 |
| Nashville (TN) | 1.28 | 1.03, 1.59 | 1.25 | 1.02, 1.54 | 1.24 | 1.00, 1.54 | 1.28 | 1.03, 1.60 | 1.24 | 1.01, 1.54 |
| North Carolina (NC) | 1.38 | 1.09, 1.75 | 1.36 | 1.07, 1.71 | 1.36 | 1.07, 1.73 | 1.35 | 1.07, 1.70 | 1.35 | 1.07, 1.72 |
| Stanford (CA) | 1.20 | 0.94, 1.53 | 1.22 | 0.95, 1.56 | 1.18 | 0.92, 1.51 | 1.13 | 0.89, 1.44 | 1.16 | 0.91, 1.48 |
| SARS-CoV-2 Vaccine receipt |  |  |  |  |  |  |  |  |  |  |
| 0-1 dose | — | — | — | — | — | — | — | — | — | — |
| 2 or more doses | 0.96 | 0.87, 1.07 | 0.96 | 0.87, 1.07 | 0.96 | 0.86, 1.06 | 0.95 | 0.86, 1.06 | 0.96 | 0.86, 1.06 |
| Race/ethnicity |  |  |  |  |  |  |  |  |  |  |
| White, Non-Hispanic | — | — | — | — | — | — | — | — | — | — |
| Asian, Non-Hispanic | 0.95 | 0.70, 1.28 | 0.95 | 0.70, 1.28 | 0.95 | 0.70, 1.29 | 0.93 | 0.69, 1.27 | 0.95 | 0.70, 1.29 |
| Black, Non-Hispanic | 0.96 | 0.80, 1.15 | 0.96 | 0.80, 1.15 | 1.00 | 0.84, 1.19 | 0.95 | 0.78, 1.14 | 1.00 | 0.83, 1.19 |
| Hispanic/Latino | 1.04 | 0.90, 1.20 | 1.04 | 0.90, 1.19 | 1.08 | 0.94, 1.23 | 1.04 | 0.90, 1.20 | 1.07 | 0.93, 1.22 |
| Multiple race, Non-Hispanic | 0.96 | 0.74, 1.24 | 0.95 | 0.74, 1.23 | 0.96 | 0.74, 1.25 | 0.96 | 0.74, 1.24 | 0.96 | 0.74, 1.25 |
| NH/OPI, Non-Hispanic | 1.52 | 1.21, 1.92 | 1.54 | 1.25, 1.89 | 1.54 | 1.25, 1.89 | 1.59 | 1.29, 1.96 | 1.51 | 1.19, 1.92 |
| Unknown/Refused | 1.20 | 0.96, 1.51 | 1.20 | 0.96, 1.50 | 1.24 | 0.99, 1.56 | 1.20 | 0.96, 1.51 | 1.24 | 0.99, 1.55 |
| missing | 0.85 | 0.62, 1.16 | 0.84 | 0.61, 1.16 | 0.86 | 0.62, 1.18 | 0.85 | 0.61, 1.17 | 0.86 | 0.62, 1.18 |

1. Adjusted models included age, sex, study site, vaccine receipt, and clustering by census tract. SARS-CoV-2 vaccine receipt defined as unvaccinated if received 1 or fewer COVID-19 vaccine doses and vaccinated if received 2 or more COVID-19 vaccine doses. Influenza vaccine receipt defined as unvaccinated if did not receive annual Influenza vaccine and vaccinated if participant did receive the seasonal Influenza vaccine.

Supplemental Table 2: Adjusted incidence risk ratios (aIRRs)^1^ of influenza among exposed household contacts by National Social Vulnerability Index overall and by domain percentiles, adjusted for age, study site, and race/ethnicity, cluster adjusted within census tract in a case ascertained household transmission study, United States, September 2021-May 2023.^2^

|  | **Overall** | | **Theme 1: Socioeconomic Status** | | **Theme 2: Household Characteristics** | | **Theme 3: Racial/ethnic minorities** | | **Theme 4: Household type-transportation-crowding** | |
| --- | --- | --- | --- | --- | --- | --- | --- | --- | --- | --- |
| **Characteristic** | **IRR***^1^* | **95% CI***^1^* | **IRR***^1^* | **95% CI***^1^* | **IRR***^1^* | **95% CI***^1^* | **IRR***^1^* | **95% CI***^1^* | **IRR***^1^* | **95% CI***^1^* |
| SVI | 1.45 | 0.88, 2.39 | 1.26 | 0.74, 2.12 | 1.52 | 0.98, 2.35 | 1.90 | 0.95, 3.80 | 1.31 | 0.81, 2.12 |
| Age | 1.00 | 0.99, 1.00 | 1.00 | 0.99, 1.00 | 1.00 | 0.99, 1.00 | 1.00 | 0.99, 1.00 | 1.00 | 0.99, 1.00 |
| Sex |  |  |  |  |  |  |  |  |  |  |
| Female | — | — | — | — | — | — | — | — | — | — |
| Male | 1.04 | 0.88, 1.22 | 1.03 | 0.87, 1.20 | 1.04 | 0.88, 1.22 | 1.03 | 0.88, 1.21 | 1.03 | 0.87, 1.21 |
| Enrollment site |  |  |  |  |  |  |  |  |  |  |
| Arizona (AZ) | — | — | — | — | — | — | — | — | — | — |
| Colorado (CO) | 1.40 | 1.17, 1.67 | 1.40 | 1.10, 1.78 | 1.51 | 1.14, 2.00 | 1.17 | 0.98, 1.39 | 1.29 | 1.10, 1.52 |
| Columbia (NY) | 0.46 | 0.31, 0.67 | 0.48 | 0.33, 0.72 | 0.49 | 0.33, 0.73 | 0.39 | 0.25, 0.61 | 0.46 | 0.31, 0.68 |
| Marshfield (WI) | 0.23 | 0.04, 1.24 | 0.23 | 0.04, 1.24 | 0.24 | 0.04, 1.35 | 0.26 | 0.05, 1.39 | 0.21 | 0.04, 1.14 |
| Nashville (TN) | 0.66 | 0.52, 0.84 | 0.64 | 0.49, 0.83 | 0.67 | 0.49, 0.91 | 0.63 | 0.50, 0.78 | 0.64 | 0.50, 0.81 |
| North Carolina (NC) | 0.69 | 0.43, 1.09 | 0.67 | 0.42, 1.09 | 0.68 | 0.41, 1.12 | 0.62 | 0.40, 0.98 | 0.65 | 0.41, 1.04 |
| Stanford (CA) | 0.85 | 0.53, 1.35 | 0.88 | 0.55, 1.42 | 0.93 | 0.56, 1.54 | 0.69 | 0.42, 1.13 | 0.81 | 0.50, 1.31 |
| Influenza Vaccine receipt |  |  |  |  |  |  |  |  |  |  |
| Unvaccinated for Influenza | — | — | — | — | — | — | — | — | — | — |
| Vaccinated for Influenza | 0.88 | 0.72, 1.07 | 0.88 | 0.72, 1.07 | 0.86 | 0.71, 1.06 | 0.88 | 0.72, 1.07 | 0.88 | 0.72, 1.07 |
| Race/ethnicity |  |  |  |  |  |  |  |  |  |  |
| White, Non-Hispanic | — | — | — | — | — | — | — | — | — | — |
| AI/AN, Non-Hispanic | 1.02 | 0.90, 1.15 | 1.00 | 0.89, 1.13 | 1.01 | 0.90, 1.14 | 1.15 | 0.95, 1.38 | 0.99 | 0.87, 1.11 |
| Asian, Non-Hispanic | 0.81 | 0.26, 2.52 | 0.80 | 0.25, 2.51 | 0.79 | 0.26, 2.37 | 0.76 | 0.27, 2.14 | 0.81 | 0.26, 2.54 |
| Black, Non-Hispanic | 0.97 | 0.63, 1.49 | 1.02 | 0.66, 1.55 | 1.00 | 0.67, 1.51 | 0.96 | 0.63, 1.47 | 1.01 | 0.66, 1.54 |
| Hispanic/Latino | 1.23 | 0.86, 1.76 | 1.27 | 0.90, 1.81 | 1.27 | 0.90, 1.78 | 1.24 | 0.87, 1.78 | 1.29 | 0.91, 1.83 |
| Multiple race, Non-Hispanic | 1.42 | 0.74, 2.70 | 1.44 | 0.74, 2.79 | 1.33 | 0.69, 2.58 | 1.31 | 0.68, 2.50 | 1.51 | 0.77, 2.98 |
| NH/OPI, Non-Hispanic | 1.58 | 1.01, 2.45 | 1.81 | 1.19, 2.76 | 1.59 | 1.08, 2.33 | 1.75 | 1.30, 2.36 | 1.74 | 1.15, 2.62 |
| Unknown/Refused | 0.94 | 0.40, 2.18 | 0.97 | 0.43, 2.20 | 0.95 | 0.41, 2.21 | 0.96 | 0.41, 2.22 | 0.98 | 0.43, 2.26 |
| missing | 0.00 | 0.00, 0.00 | 0.00 | 0.00, 0.00 | 0.00 | 0.00, 0.00 | 0.00 | 0.00, 0.00 | 0.00 | 0.00, 0.00 |

| Unknown/Refused | 1.20 | 0.96, 1.51 | 1.20 | 0.96, 1.50 | 1.24 | 0.99, 1.56 | 1.20 | 0.96, 1.51 | 1.24 | 0.99, 1.55 |
| --- | --- | --- | --- | --- | --- | --- | --- | --- | --- | --- |
| missing | 0.85 | 0.62, 1.16 | 0.84 | 0.61, 1.16 | 0.86 | 0.62, 1.18 | 0.85 | 0.61, 1.17 | 0.86 | 0.62, 1.18 |

1. Adjusted models included age, sex, study site, vaccine receipt, and clustering by census tract. SARS-CoV-2 vaccine receipt defined as unvaccinated if received 1 or fewer COVID-19 vaccine doses and vaccinated if received 2 or more COVID-19 vaccine doses. Influenza vaccine receipt defined as unvaccinated if did not receive annual Influenza vaccine and vaccinated if participant did receive the seasonal Influenza vaccine.

**Supplemental Table 3:** Mediation analysis using the Baron-Kenny method for SARS-CoV-2 households

| SARS-CoV-2 household contacts |  |  |
| --- | --- | --- |
| Regressing Household infection on SVI (Y on X) |  |  |
| Characteristic | **IRR***^1^* | **95% CI***^1^* |
| **SVI: overall** | **1.20** | **1.04, 1.38** |
| **SVI: Socioeconomic status** | **1.24** | **1.08, 1.43** |
| SVI: Household characteristics | 1.11 | 0.95, 1.30 |
| **SVI: Racial/ethnic minorities** | **1.25** | **1.05, 1.48** |
| SVI: Household type, transportation, crowding | 1.12 | 0.97, 1.30 |
| Regressing vaccination receipt (yes/no) on SVI (M on X) |  |  |
| Characteristic | **IRR***^1^* | **95% CI***^1^* |
| **SVI: overall** | **0.88** | **0.83, 0.92** |
| **SVI: Socioeconomic status** | **0.87** | **0.83, 0.92** |
| **SVI: Household characteristics** | **0.87** | **0.83, 0.92** |
| **SVI: Racial/ethnic minorities** | **0.93** | **0.88, 0.99** |
| **SVI: Household type, transportation, crowding** | **0.89** | **0.85, 0.93** |
| Regressing household infection on SVI + vaccine receipt (Y on X+M) |  |  |
| Characteristic | **IRR***^1^* | **95% CI***^1^* |
| **SVI: overall** | **1.18** | **1.02, 1.36** |
| Vaccine receipt |  |  |
| No | Reference |  |
| Yes | 0.93 | 0.84, 1.02 |
| **SVI: Socioeconomic status** | **1.22** | **1.06, 1.41** |
| Vaccine receipt |  |  |
| No | Reference |  |
| Yes | 0.93 | 0.85, 1.03 |
| SVI: Household characteristics | 1.09 | 0.93, 1.27 |
| Vaccine receipt |  |  |
| No | Reference |  |
| Yes | 0.91 | 0.83, 1.01 |
| **SVI: Racial/ethnic minorities** | **1.23** | **1.04, 1.46** |
| Vaccine receipt |  |  |
| No | Reference |  |
| Yes | 0.92 | 0.83, 1.01 |
| SVI: Household type, transportation, crowding | 1.1 | 0.95, 1.28 |
| Vaccine receipt |  |  |
| No | Reference |  |
| Yes | 0.92 | 0.83, 1.01 |

**Supplemental Table 4:** Mediation analysis using the Baron-Kenny method for influenza households

| Influenza household contacts |  |  |
| --- | --- | --- |
| Regressing Household infection on SVI (Y on X) |  |  |
| **Characteristic** | **IRR***^1^* | **95% CI***^1^* |
| SVI: Overall | 1.17 | 0.88, 1.56 |
| SVI: Socioeconomic status | 1.14 | 0.84, 1.54 |
| SVI: Household Characteristics | 1.27 | 0.92, 1.77 |
| SVI: Racial/ethnic minorities | 1.25 | 0.87, 1.79 |
| SVI: Household type, transportation, crowding | 1.15 | 0.85, 1.56 |
| Regressing influenza vaccination receipt (yes/no) on SVI (M on X) |  |  |
| **Characteristic** | **IRR***^1^* | **95% CI***^1^* |
| **SVI: Overall** | **0.90** | **0.81, 1.00** |
| **SVI: Socioeconomic status** | **0.89** | **0.79, 1.00** |
| SVI: Household Characteristics | 0.97 | 0.86, 1.10 |
| **SVI: Racial/ethnic minorities** | **0.86** | **0.76, 0.98** |
| **SVI: Household type, transportation, crowding** | **0.88** | **0.78, 0.98** |
| Regressing household infection on SVI + Influenza vaccine receipt (Y on X+M) |  |  |
| **Characteristic** | **IRR***^1^* | **95% CI***^1^* |
| SVI: Overall | 1.14 | 0.85, 1.53 |
| Vaccine receipt |  |  |
| No | Reference |  |
| Yes | 0.87 | 0.70, 1.06 |
| SVI: Socioeconomic status | 1.11 | 0.82, 1.51 |
| Vaccine receipt |  |  |
| No | Reference |  |
| Yes | 0.86 | 0.70, 1.06 |
| SVI: Household Characteristics | 1.27 | 0.91, 1.76 |
| Vaccine receipt |  |  |
| No | Reference |  |
| Yes | 0.86 | 0.70, 1.05 |
| SVI: Racial/ethnic minorities | 1.21 | 0.84, 1.75 |
| Vaccine receipt |  |  |
| No | Reference |  |
| Yes | 0.87 | 0.71, 1.07 |
| SVI: Household type, transportation, crowding | 1.12 | 0.82, 1.52 |
| Vaccine receipt |  |  |
| No | Reference |  |
| Yes | 0.86 | 0.70, 1.06 |

## Supplemental Figures

**Supplemental Figure 1: CONSORT Diagram for SARS-CoV-2 cohort inclusion and exclusion criteria**


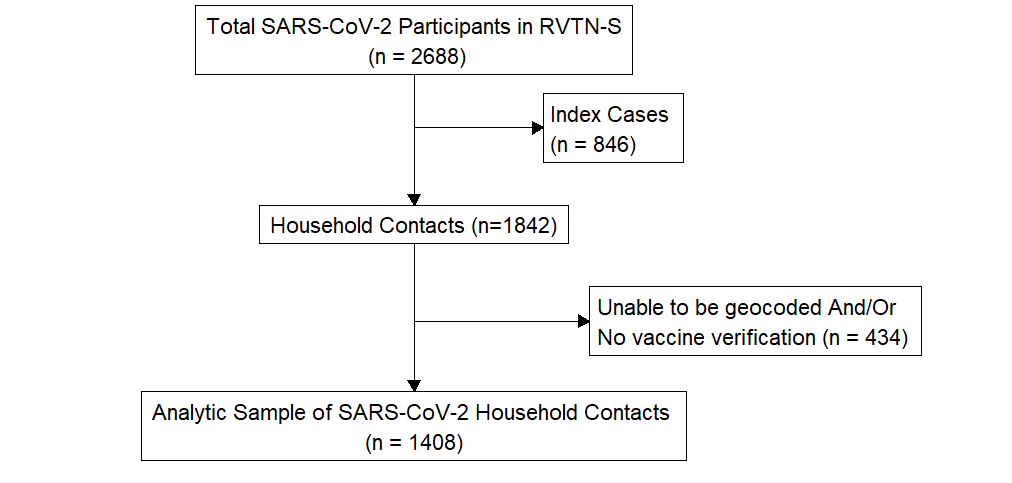


**Supplemental Figure 2: CONSORT Diagram for influenza cohort inclusion and exclusion criteria**
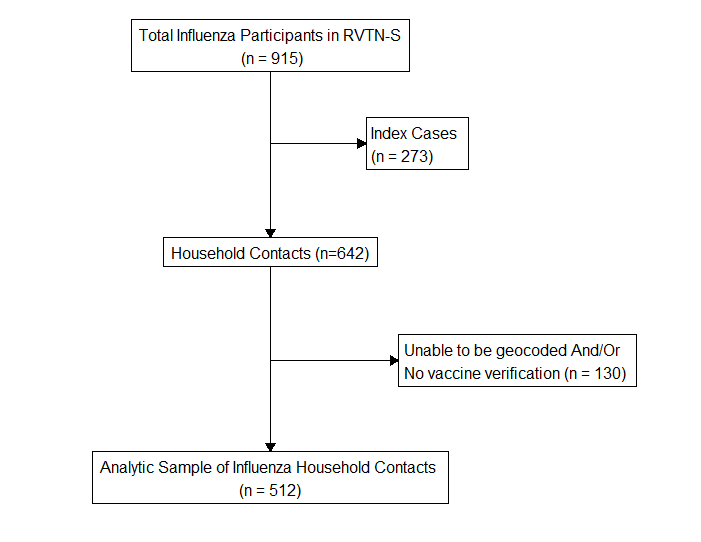


**Supplemental Figure 3:** Box and Whisker plots of SVI domain percentile at the census tract level by vaccine receipt^1^ among SARS-CoV-2 household contacts.


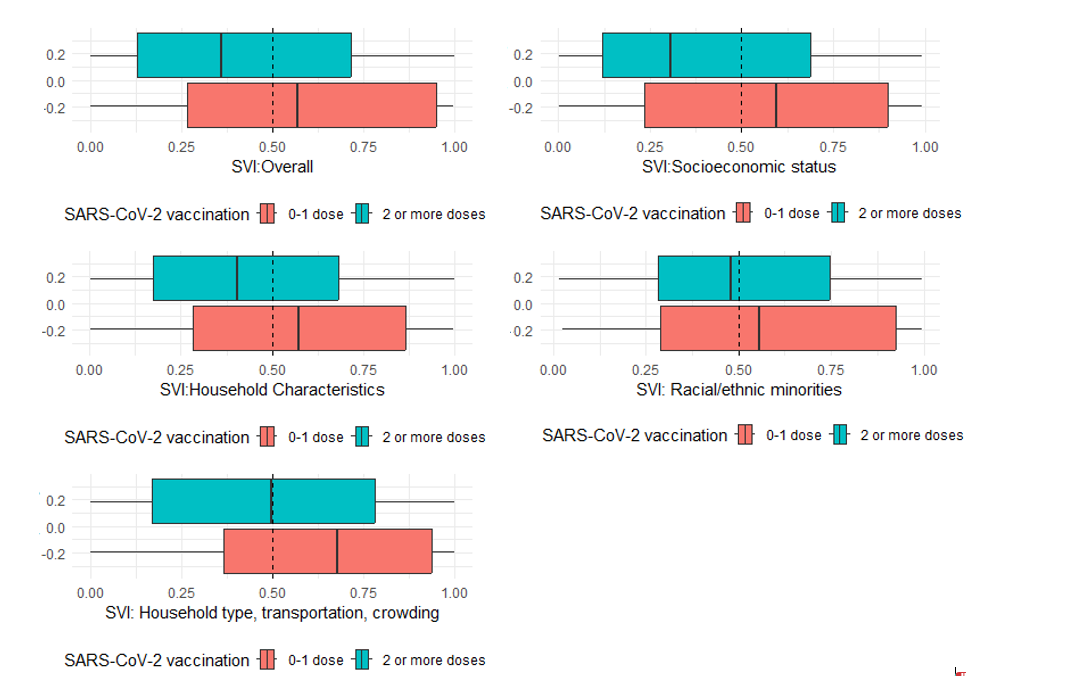


Footnote:

^1^ SARS-CoV-2 vaccine receipt defined as unvaccinated if received 1 or fewer COVID-19 vaccine doses and vaccinated if received 2 or more COVID-19 vaccine doses.

**Supplemental Figure 4:** Box and Whisker plots of SVI domain percentile at the census tract level by vaccine receipt^1^ among the Influenza household contacts.


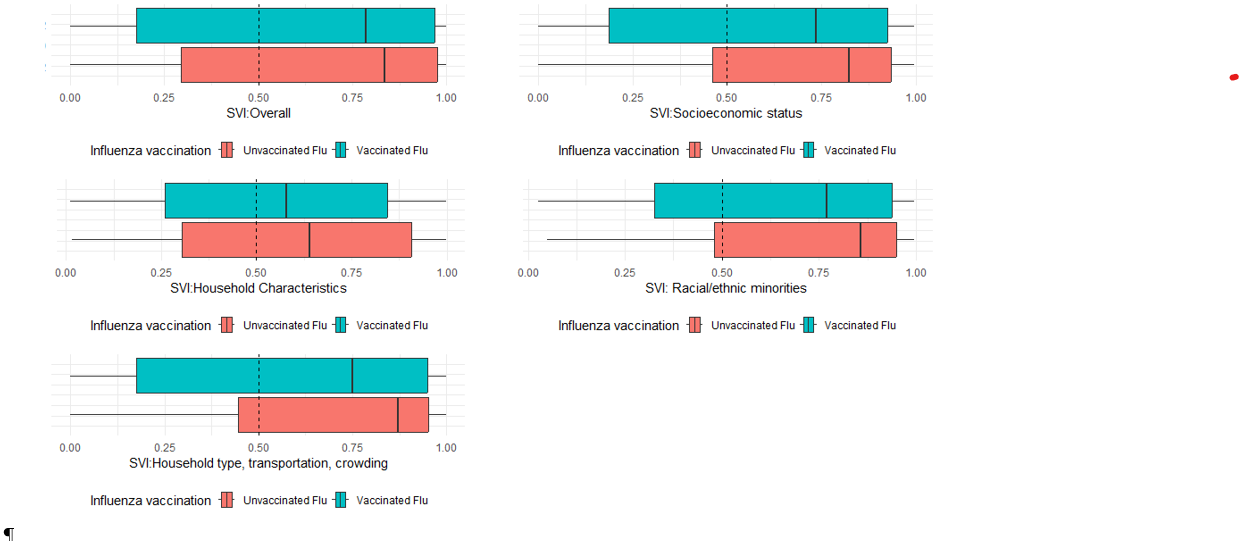


Footnote:

^1^ Influenza vaccine receipt defined as unvaccinated if did not receive annual Influenza vaccine and vaccinated if participant did receive the seasonal Influenza vaccine.

Supplemental Figure 5: **Forest plots of** Crude (cIRR) and adjusted Incidenc Risk Ratios (aIRRs)^1^ of SARS-CoV-2 or Influenza among exposed household contacts by National or State Social Vulnerability Index overall and by domain percentiles, adjusted for age, sex, study site, race/ethnicity, and vaccine receipt. cluster adjusted within census tract in a case ascertained household transmission study, United States, September 2021-May 2023


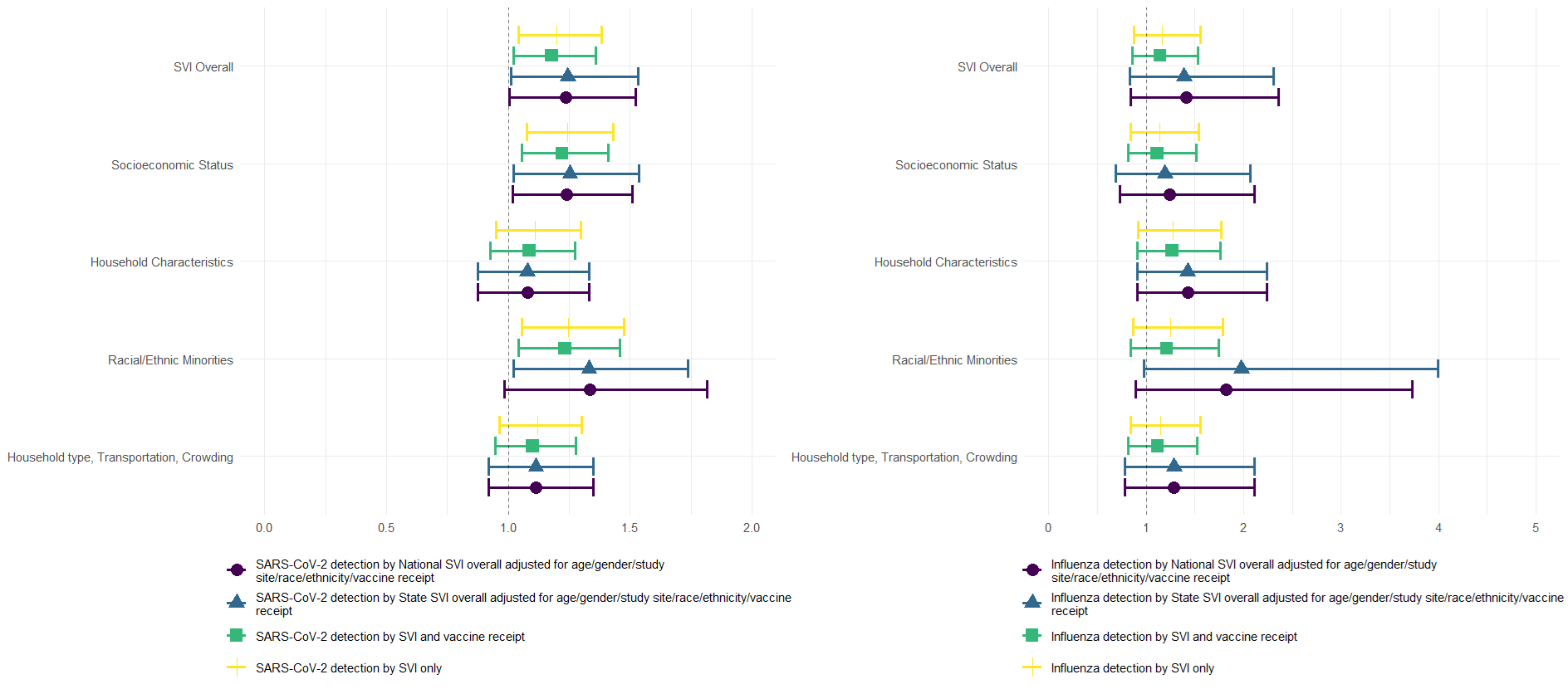


^1^ Adjusted models included age, sex, study site, vaccine receipt, and clustering by census tract. SARS-CoV-2 vaccine receipt defined as unvaccinated if received 1 or fewer COVID-19 vaccine doses and vaccinated if received 2 or more COVID-19 vaccine doses. Influenza vaccine receipt defined as unvaccinated if did not receive annual Influenza vaccine and vaccinated if participant did receive the seasonal Influenza vaccine.
